# Biomedical diagnosis perspective of epigenetic detections using alpha-hemolysin nanopore

**DOI:** 10.3934/matersci.2015.4.448

**Published:** 2015-11-16

**Authors:** Yong Wang, Li-qun Gu

**Affiliations:** Department of Biological Engineering, Dalton Cardiovascular Research Center, University of Missouri-Columbia, Columbia, MO 65211, USA

**Keywords:** α-hemolysin, microRNA, G-quadruplex, DNA damage, guanine oxidation, abasic site, DNA methylation, DNA hydroxymethylation, nanopore device, cancer biomarker

## Abstract

The α-hemolysin nanopore has been studied for applications in DNA sequencing, various single-molecule detections, biomolecular interactions, and biochips. The detection of single molecules in a clinical setting could dramatically improve cancer detection and diagnosis as well as develop personalized medicine practices for patients. This brief review shortly presents the current solid state and protein nanopore platforms and their applications like biosensing and sequencing. We then elaborate on various epigenetic detections (like microRNA, G-quadruplex, DNA damages, DNA modifications) with the most widely used alpha-hemolysin pore from a biomedical diagnosis perspective. In these detections, a nanopore electrical current signature was generated by the interaction of a target with the pore. The signature often was evidenced by the difference in the event duration, current level, or both of them. An ideal signature would provide obvious differences in the nanopore signals between the target and the background molecules. The development of cancer biomarker detection techniques and nanopore devices have the potential to advance clinical research and resolve health problems. However, several challenges arise in applying nanopore devices to clinical studies, including super low physiological concentrations of biomarkers resulting in low sensitivity, complex biological sample contents resulting in false signals, and fast translocating speed through the pore resulting in poor detections. These issues and possible solutions are discussed.

## 1. Introduction

### 1.1. Epigenetics and its detections

The term epigenetics was introduced by Conrad Waddington in the early 1940s. Originally, epigenetics referred to the molecular pathways modulating the expression of a genotype into a particular phenotype, but the meaning of the term has gradually narrowed over the decades [1]. Unlike genetics studies based on alterations to the DNA sequence (the genotype), the epigenetics refer to the changes in gene expression or cellular phenotype which have other causes. Epigenetics also refers to the changes to the genome that do not involve a change in the nucleotide sequence. Examples of mechanisms that produce such changes are DNA methylation, loss of imprinting and histone modification, each of which affects how genes are expressed but without altering the underlying DNA sequence.

Epigenetic are affected by many factors like development, environmental chemicals, diet, drugs or pharmaceuticals, aging, which can cause cancer, autoimmune disease, mental disorder, diabetes and so on. Epigenetic modulations are fundamental to the genesis of cancer [2–4]. Detection of epigenetic alterations as biomarkers for cancer detection, diagnosis and prognosis have been studied extensively and were advanced rapidly [5–7].

### 1.2. Nanopore

A nanopore is a small hole, with the diameter from several nanometers (nm) to hundreds of nanometers. It could be a pore-forming protein, ion channels (biological nanopores), or a hole in synthetic materials such as glass, silicon, silicon nitride or graphene (solid state nanopores). Solid state nanopores are generally made in silicon membranes, the most common being silicon nitride [8–10]. Others like graphene [11–13], glass [14, 15] or glass slides [16] are used, as well. Solid state nanopores can be manufactured with several techniques including ion-beam sculpting [17], the transmission electron microscope (TEM) technique, or by electron beams [18]. Current biological nanopore platforms include the most widely used alpha-hemolysin [19], and others like MspA [20,21], aerolysin [22], OccK channels [23], FhuA [24], β-barrel protein nanopore [25], SP1 [26], Phi29 [27] and Cytolysin [28]. Composing hybrid nanopores is a newly evolved research field that began around 2007. Researchers are working on functionalizing solid state nanopores with biologically compatible polymer coatings and inserting biological-forming pores into the solid state nanopores, which will make the hybrid nanopores available for many different applications [29]. Studies have found that pre-assembled aHL protein pore can be inserted into a silicon nitride membrane with small holes, of diameters from 2.4–3.6 nm [30]. Many research groups are working on coating specific recognition sequences and receptors to nanopores, chemical functionalization of solid nanopores, like DNA Origami Nanopores [31], functionalized Solid Nanopores [32–35], and biofriendly nanochannels in a thin solid membrane [36].

### 1.3. Nanopore applications

Utilizing the electrophysiology method, i.e., patch clamp single channel recording technology, the ion current through a nanopore is very sensitive to the target molecules when occupying and translocating the pore. The interactions of the molecules and the pore can generate characteristic change in the nanopore current, therefore forming nanopore signatures (current-time relationship) as well as different molecular states which can be electrically identified.

The nanopores have been extensively studied for genetic detections [37–40], epigenetic detections [20,41–43], single-molecule detections [44–49], and biomolecular interactions [50–56] and are now being developed for next-generation sequencing [57–59].

This review will focus on epigenetic detections with the most widely used alpha-hemolysin nanopore (α-HL). Significance regarding biological functions and medicinal research of these epigenetic detections will also be reviewed and discussed.

## 2. microRNA Detections

In humans, microRNA genes represent approximately 1% of genome but regulate approximately 30% of human genes [60]. MicroRNAs are a group of small non-coding cellular RNAs with important biological functions in regulation of cell development, differentiation, apoptosis, and signaling pathways [61]. By systematic analysis of microRNA expression profiles, microRNA “signatures” have been identified in different types of cancers. Furthermore, cancer cells release certain microRNAs into circulation. These blood microRNAs are present in a very stable format and have thus been considered as potential biomarkers for cancer detection [62–64].

### 2.1. Detection of circulating microRNAs in cancer patients

**Figure 1.**
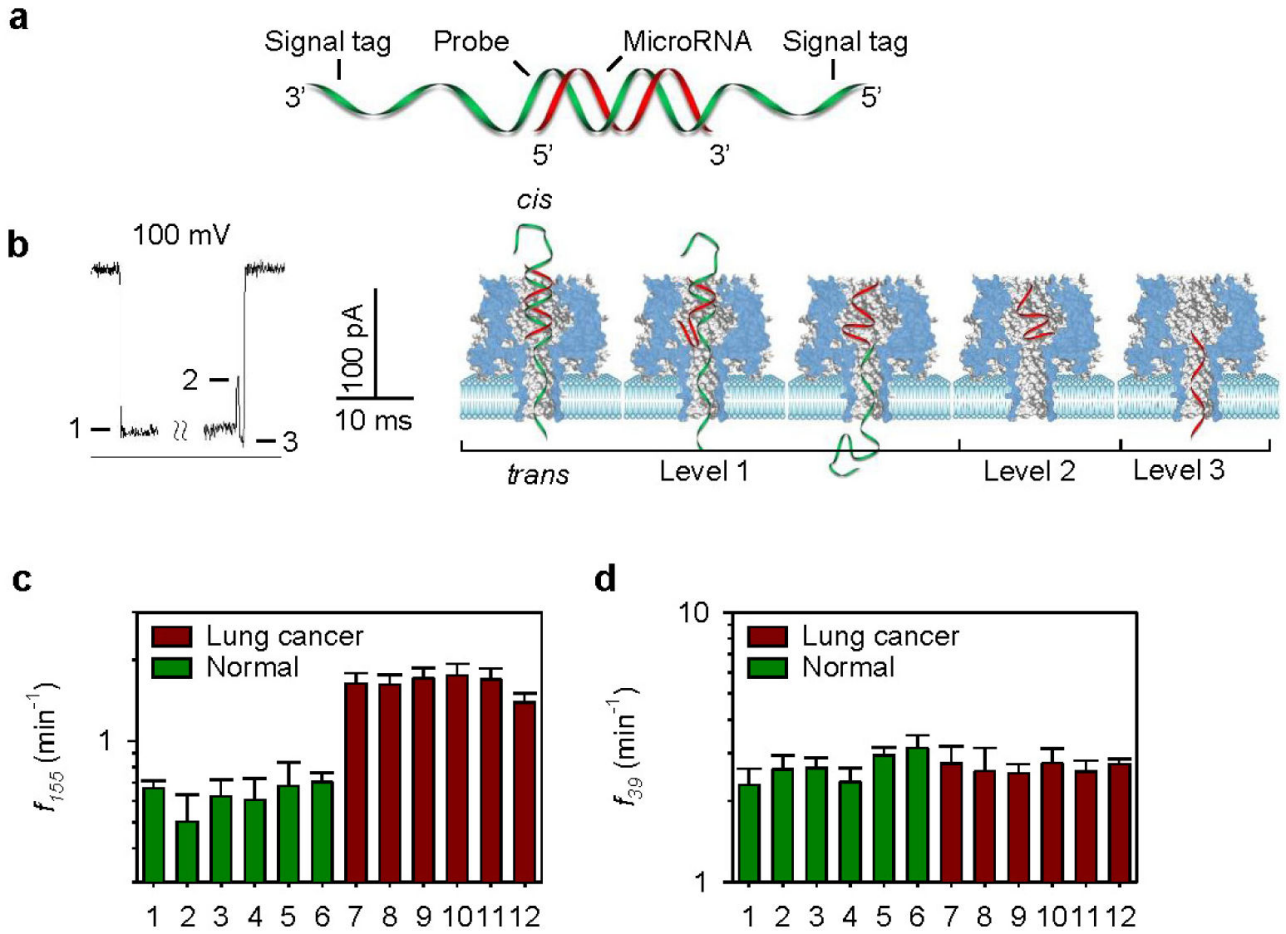
microRNA detections in the lung cancer patients. **a,** Molecular diagram of a microRNA (red) bound to a probe (green) with signal tags. **b,** The unzipping signature (left) and the molecule pathways of the dsDNA unzipping signature (right) in the nanopore. **c,** The frequencies of *miR-155* signature events (*f_155_*) from six healthy individuals (#1 to #6) and six patients with lung cancer (#7 to #12) in the presence of spiked-in synthetic *miR-39*. **d** The frequencies of spike-in *miR-39* signature events detected all the samples that were used in c. The patient conditions were the following: #7, metastatic squamous lung carcinoma; #8, recurrent small-cell cancer; #9, early-stage small-cell carcinoma, status post-chemotherapy and -radiation; #10, early-stage small-cell cancer, status post-chemotherapy; #11, late-stage non-small cell carcinoma, status post-resection and -chemotherapy; #12, late-stage adenocarcinoma, status post-chemotherapy. Reprinted with permission from reference [65].

It is difficult to distinguish the translocation of different microRNAs in α-HL because the sequences of all microRNAs are short and similar in length. One way to overcome this challenge is to use a signature that can detect target microRNA in the mixture. Studies have identified such a microRNA signature signal in the α-HL using an oligonucleotide probe with a signal tag (Figure 1 a, b). The analysis indicated two important functions performed by the signal tag of the probe: 1) guidance of the microRNA**·**probe complex entrapment in the pore and 2) inducement of the dissociation of the microRNA**·**probe complex. The configuration change during the unzipping process gave rise to signature current patterns, which enabled the recognition of single target microRNA molecules. Because of the specificity of the probe, the frequency of the signature signal was independent of the presence of multiple nucleic acid components and could therefore be used to quantify target microRNA in the mixture.

The level of mir-155 in blood samples from cancer patients was significantly higher than the normal healthy controls, but the level of spiked-in internal control of mir-39 (synthetic C. elegans microRNA, not presented in human) remained unchanged in these samples (Figure 1 c, d). Overall, the signature signal ensured the high selectivity required for microRNA detection in plasma RNA extract [65]. The key component of the nanopore sensor is the probe, the sequence of which is programmable and can be optimized to achieve high sensitivity and selectivity. The nanopore method can be a useful tool for quantitative studies of microRNAs and the discovery of disease markers, which are important for non-invasive screening and the early diagnosis of diseases such as cancer.

### 2.2. Developing a novel polycationic probe for simultaneous enrichment and detection of MicroRNAs

Generally, the clinical samples used to test for microRNA are RNA extractions from a patient’s biofluids such as plasma. These extractions are a complex collection of various RNA species: miRNAs, mRNAs, tRNAs, etc. When the nanopore is used to detect the target miRNA, any free nucleic acids in the RNA mixture can also nonspecifically interact with the pore. These interactions can result in “contaminative” signals in the α-HL that severely influence the target miRNA determination, and these signals should be eliminated.

**Figure 2.**
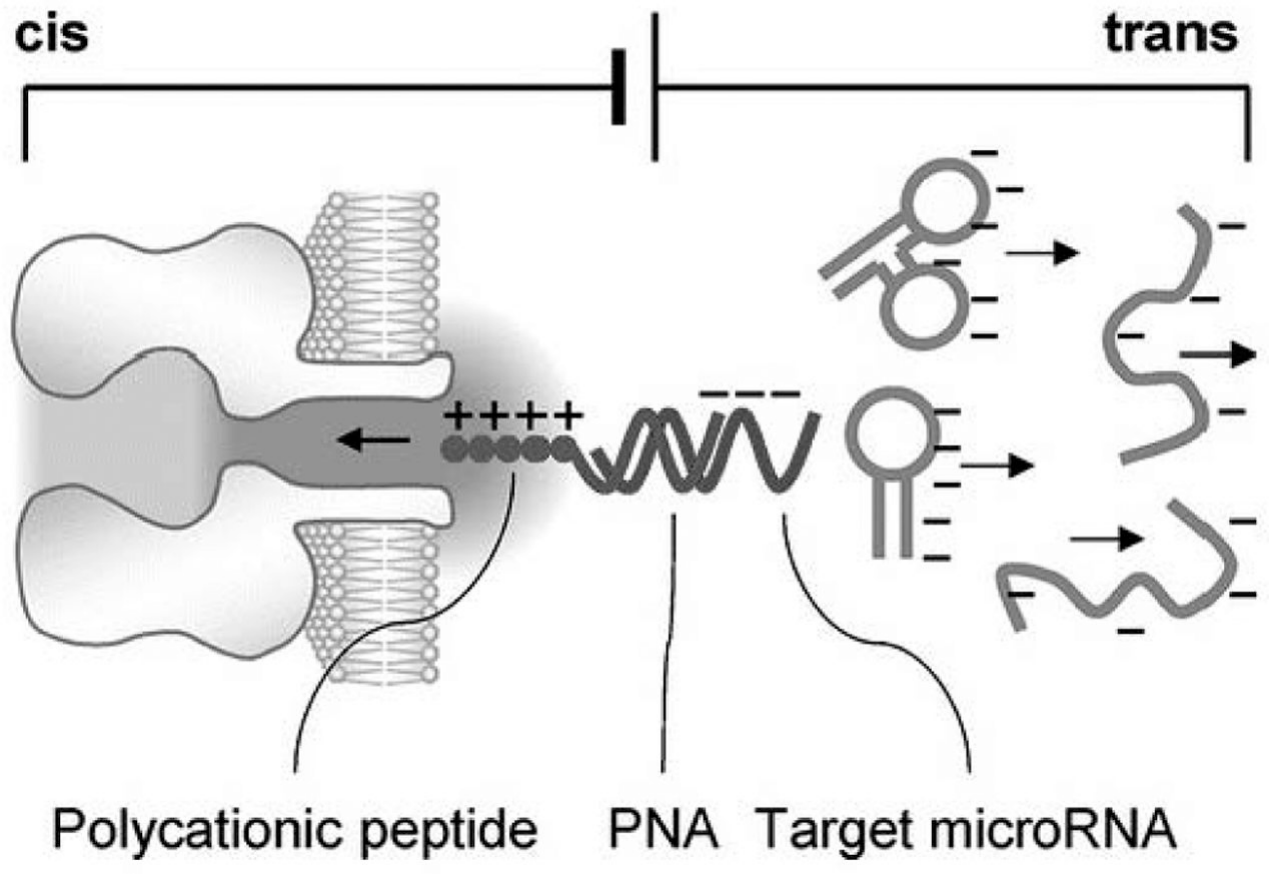
Cationic peptide probe-based interference-free detection of microRNAs in the nanopore. The probe includes: a polycationic polymer lead (peptide, blue) and a capture domain (PNA, green). The capture domain can hybridize with the target microRNA (red). The miRNA**·**probe complex is drawn into the nanopore by the applied voltage at the pore (trans) opening, while all other negatively charged free nucleic acids without the probe binding (gray) were electrophoretically expelled away from the pore. Reprinted with permission from reference [66].

By using a polycationic probe as the carrier, the nanopore can selectively capture and detect the target miRNA. The probe comprises a sequence of peptide nucleic acids (PNA) conjugated with a polycationic peptide lead. The PNA is designed to specifically capture the target miRNA. Upon hybridization, the positively charged peptide lead and the negatively charged miRNA together form a dipole (Figure 2). This structure can be driven into the nanopore by a large electric field gradient around the nanopore opening. At the same time, any free nucleic acids without probe hybridization would carry negative charge and migrate away from the pore opening. Consequently, only the signatures for the miRNA**·**probe complex and probe alone in the nanopore will be identified, and any interference signal originating from free nucleic acids is completely eliminated [66]. This method allows researchers to selectively detect only those nucleic acid sequences that hybridize with the probe, even when many other confounding species are present. If validated in clinical samples, for example, the detection of target miRNA from RNA extractions derived from a patients’ biofluids, this method would have applications in many areas such as early disease diagnosis, cancer metastasis prediction, and the monitoring of a patient’s response to therapy. In conclusion, this novel approach introduces a new method of detecting clinically relevant DNA or RNA fragments in a complex nucleic acid mixture.

### 2.3. Developing probes for multiplex microRNA detection

Cancer/disease detection and diagnosis require accurate measurement of a biomarker panel, rather than a single miRNA species. The current nanopore technology cannot meet this need because it can analyze only one miRNA *per* detection. Although nanopore multiplex detection has been reported, their common principle, *i.e.*, different targets (unlabeled or labeled) generating distinct nanopore signatures, is not applicable to miRNA detection. Due to their similar polymer lengths (18–22 bases), miRNAs cannot be distinguished from each other by their signatures. This demands new nanopore strategies for multiplex biomarker detection.

**Figure 3.**
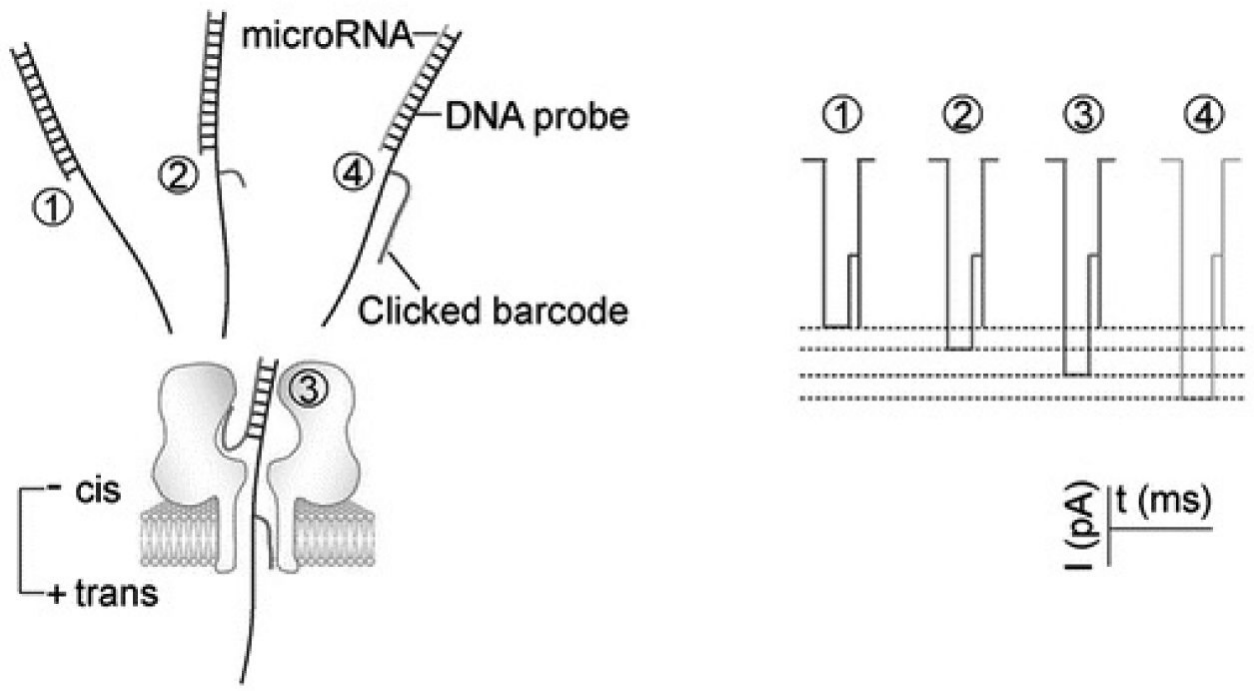
Barcode tagged probe through click chemistry for nanopore ionic current modulation and multiplex detection of mircroRNAs in the nanopore. Each PEG-labeled DNA probe can target a specific microRNA and generate a specific nanopore current profile. Upon being captured in the nanopore, the PEG on the probe specifically regulates the nanopore current profile, thereby generating a signature for target identification. Reprinted with permission from reference [68].

Studies have designed a series of barcode probes that were constructed through click chemistry (Figure 3). Each barcode motif trapped in the nanopore can specifically modulate the nanopore ionic current and therefore can encode different target nucleic acids. These universal barcode probes working together enable simultaneous detection of multiple miRNAs in a biomarker panel [67]. The target was a panel of four lung cancer-derived miRNAs: miR-155, miR-182-5p, miR-210, and miR-21. For each miRNA, four probes were constructed: one without a tag (P0) and the other three tagged with a 3-, 8-, and 24-mer PEG [68] (P3, P8, and P24) on the lead, respectively. The PEG label allows generating a distinct current profile compared with the block using unlabeled probe, and modulation of miRNA·probe blocking level by PEGs of different lengths were successful. Simultaneous observation of multiple miRNA·probe blocking levels in a current trace was also achieved. This method elucidated a biophysical mechanism for modulating nanopore ionic flow through tagging a barcode motif on the nucleic acid duplex. The barcode tag sliding in the pore marked the molecular processes of trapping, unzipping, and translocation. As each barcode tag specifically blocked the nanopore, different barcodes could be used to encode different target sequences, therefore realizing nanopore multiplex detection.

## 3. G-quadruplex Folding and Detections

Repetitive DNA sequences (telomere sequence) located at the ends of chromosomes can fold into compact structures called G-quadruplexes (also known as G-tetrads or G_4_-DNA). They are rich in guanine and are capable of forming a four-stranded structure. The quadruplex structure is further stabilized by the presence of a cation, like potassium. G-quadruplex plays an important role in epigenetics (replication, recombination, DNA repair, maintaining genome stability) and regulation processes, as well as having the potential to serve as the drug target of G-quadruplex binding proteins [69–73].

### 3.1. Folding and unfolding of G-quadruplex subtypes in a confined space

**Figure 4.**
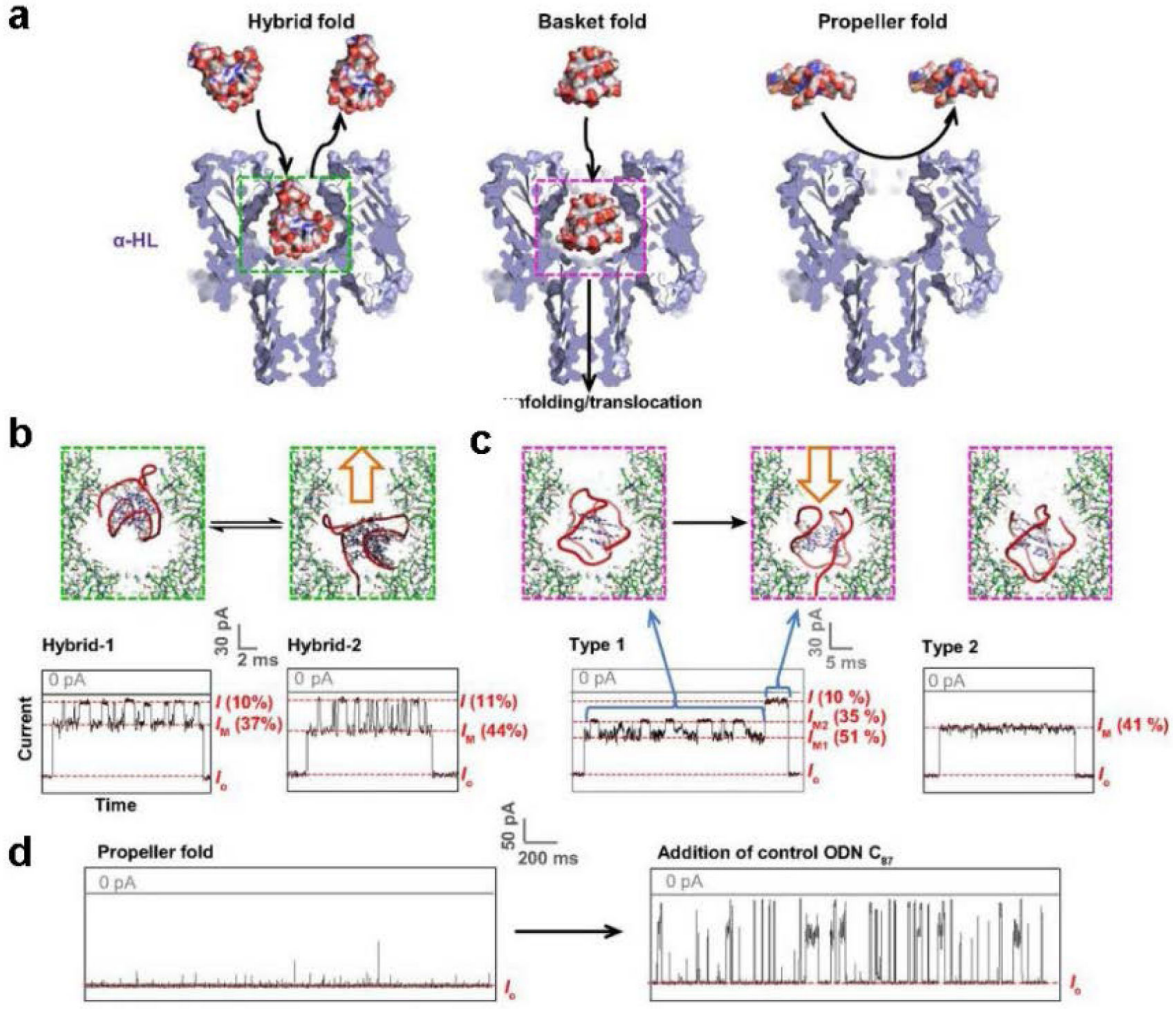
Interactions between G-quadruplex [5’-TAGGG(TTAGGG)_3_TT-3’] subtypes and the alpha-hemolysin reveal the size-dependent properties of the protein nanopore. **a,** Space-filling models of G-quadruplexes interacting with the nanopore constructed using different PBD structures, **b-d,** Stick models of the proposed interaction mechanisms and *current-time* traces yielded by (**b**) the hybrid folds (50 mM KCl, 950 mM LiCl, 25 mM Tris, pH 7.9), (**c**) the basket folds (1 M NaCl, 25 mM Tris, pH 7.9), and (**d**) the propeller folds (20 mM KCl, 5 M LiCl, 25 mM Tris, pH 7.9). All current traces were recorded under 120 mV (*trans* vs. *cis*). *I* and *I_M_* values are indicated as a percentage of the open-channel current *I_o_*. Reprinted with permission from reference [75].

A particular study demonstrated that various G-quadruplex conformations (hybrids, basket, and propeller folds) adopted by the human telomeric sequence are formed under different physical conditions, and these conformations were identified. These subtype conformations were found to form characteristic current patterns in the nanopore (Figure 4).

The basket-folded structure was able to unravel within the nanopore, while the hybrid folds had much more difficulty unfolding. In contrast, the propeller fold could not enter the nanopore opening because of its disk-like shape and larger dimensions; however, it could unfold outside the protein vestibule much faster than the basket fold. This study demonstrated the ability of the nanopore to discriminate different G-quadruplex secondary structures based on their specific shapes and sizes, as well as monitoring their unfolding kinetics at different locations (pore opening, nanocavity, constriction site) in the nanopore, thus expanding the applications of nanopore technology [74].

As we described above, the human telomere sequence (repetitive 5’-TTAGGG-3’) can fold into G-quadruplexes with different secondary structures. Hybrid folds with triplex folding intermediates can generate structure-dependent electrical current signatures within the nanocavity of the α-HL [75]. This telomere sequence is hypersensitive to UV-induced thymine dimer (T=T) formation. Studies have demonstrated that the generation of T=T only slightly changes the stabilities of hybrid and basket folds, and the presence of the T=T pair changed the ratio of the hybrid types in the hybrid folds [76].

### 3.2. Folding and unfolding of thrombin-binding aptamer formed G-quadruplex in electrolytes with different cations

**Figure 5.**
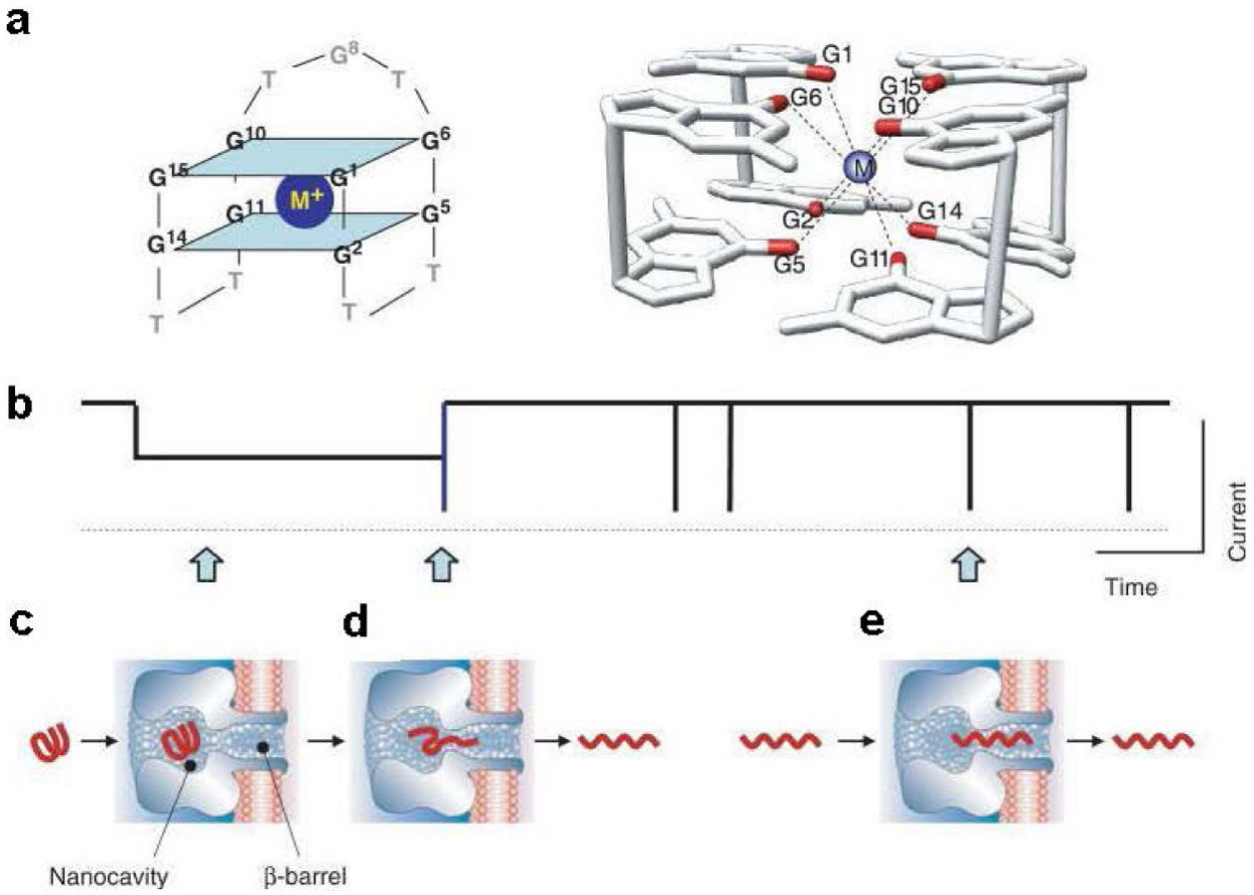
Detection of thrombin-binding aptamer (TBA, GGTTGGTGTGGTTGG) with metal ions in the nanopore. **a,** the sequence and structure of TBA G-quadruplex (left) and the two G-tetrad planes in the TBA G-quadruplex formed by guanines at the position 1, 6, 10 and 15, and the bottom one by guanine 2, 5, 11 and 14 (right). A cation in between is coordinated with eight carbonyls. **b,** Schemes of the current trace showing characteristic signature blocks. **c,** Long-lived block for capturing a single G-quadruplex in the nanocavity enclosed by the α-hemolysin nanopore; **d,** The long block terminal spike produced by translocation of the unfolded G-quadruplex through the β-barrel. The long-lived block with an ending spike served as the nanopore signature for the folded form of TBA. **e,** Short-lived block formed by translocation of linear form TBA. Reprinted with permission from reference [78].

G-quadruplexes play important roles in both gene regulation and nanosensor constructions. Studies found that cations can regulate the folding and unfolding of the G-quadruplex formed by the thrombin-binding aptamer (TBA, GGTTGGTGTGGTTGG). Single G-quadruplexes can be trapped in the nanocavity of the nanopore. The trapped Single G-quadruplexes trapped in the nanocavity of the nanopore specifically blocked the current through the nanopore [77]. The nanopore electrical signatures (Figure 5) revealed that the G-quadruplex formation was regulated by the type of cations. Cations like K^+^, Ba^2+^ and NH_4_^+^ were more favorable over Cs^+^, Na^+^ and Li^+^ for G-quadruplexes. Meanwhile, cations like Mg^2+^ and Ca^2+^ did not induce G-quadruplex formation [78]. The nanopore was also demonstrated to be efficient for the study of interactions with the protein ligand [79].

This study demonstrated that α-HL is a useful single-molecule tool for studying specific molecular processes like the ion-regulated oligonucleotides. The method used in this study may be expanded for the kinetic study of other quadruplexes and their variants. Potential targets include various biologically relevant intramolecular quadruplexes, such as the i-motif (quadruplexes formed by cytidine-rich sequences) and chemically modified quadruplexes with unique functionalities. This research may also be helpful in constructing new molecular species with tunable properties for nano-constructions and the manufacture of biosensors [79].

## 4. Detection of DNA Damages

DNA damage can happen in a variety of ways, which can ultimately lead to mutations and genomic instability without a means of repair. This might result in the development of a variety of cancers. Damage can be caused by the environment (like excessive exposure to UV radiation in the form of sunlight or tobacco smoke), oxidative damage (like free radicals), error in DNA replication, or the loss of DNA bases known as AP (apurinic/apyrimidinic) sites. Developing different kinds of biosensors for DNA damage detection has been extensively studied recently [80–83]. Below, the alpha-hemolysin based sensors for detecting DNA damages are summarized and discussed.

### 4.1. Detection of guanine oxidation in the human telomere repeat sequence

8-oxo-7,8-dihydroguanine (OG) produced from guanine (G) oxidation is a biomarker of oxidative stress which could induce telomere shortening and cellular senescence. Firstly, the natural forms of G and OG in the human telomere sequence (hTelo, 24-mer) at different locations (top tetrad, middle tetrad and bottom tetrad) were studied in the nanopore, and the singly oxidized G4 system can be discriminated from the natural form based on the event durations. However, it is limited in the clinical studies since a telomere from a cellular source will possess ~10^4^ 5’-TTAGGG-3’ repeats that can fold to series of ~10^3^ G4s, leading to the event durations being very broad. It then will not be able to distinguish the events generated by the G and OG strands in the nanopore [84].

**Figure 6.**
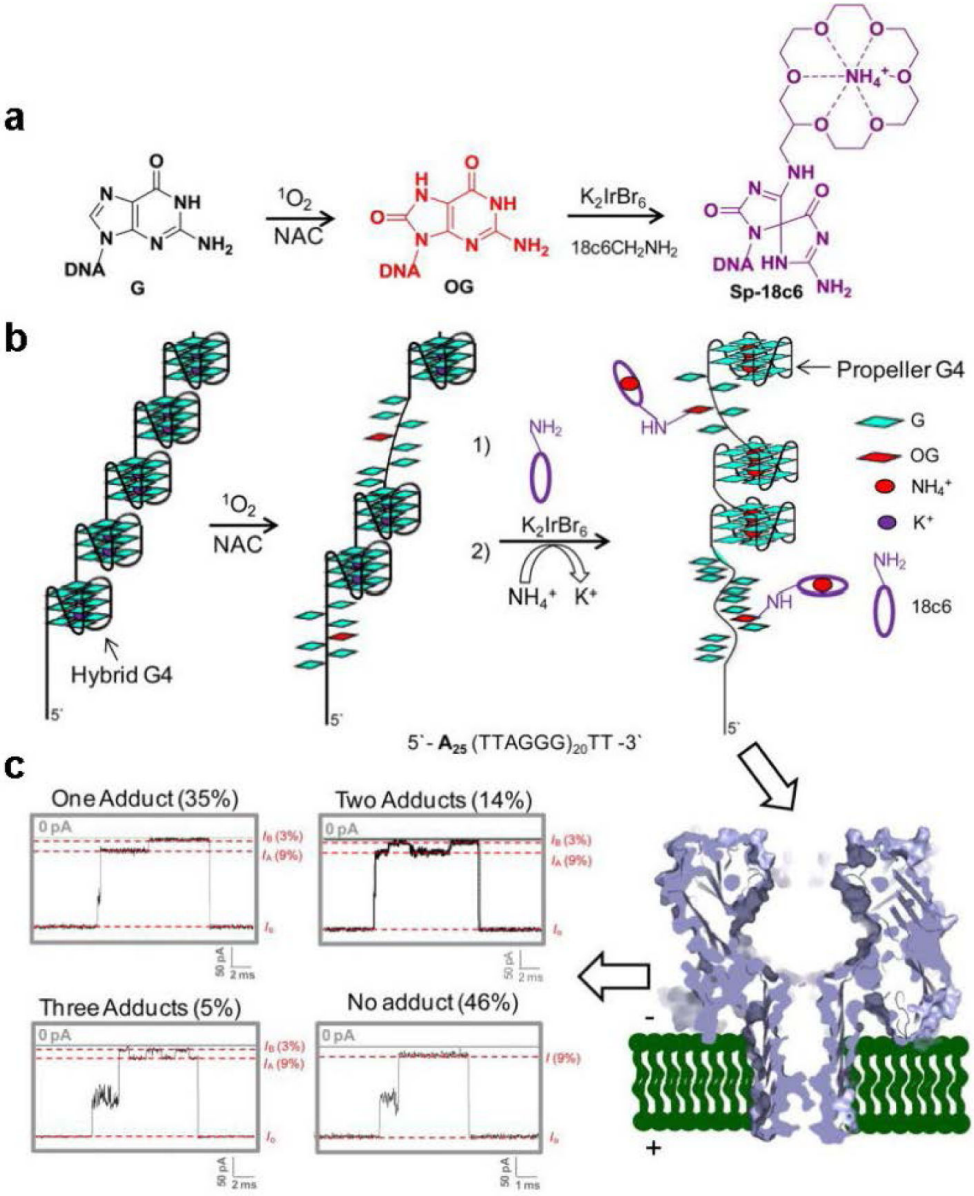
Detection of guanine oxidation in the human telomere repeat sequence. Oxidation of a 120-mer portion of the human telomere repeat sequence (Q5) with ^1^O_2_ to yield 8-oxo-7,8-dihydroguanine (OG) that was labeled with 18c6 followed by nanopore detection and quantification. **a,** Reaction scheme for oxidation of guanine to yield OG and the labeling reaction of OG by 18c6 in the presence of K_2_IrBr_6_. **b,** Model of the Q5 strand in biologically relevant salts (hybrid G4) followed by oxidation labeling and refolding in NH_4_Cl (100 mM) and LiCl (2 M) electrolyte to yield the propeller fold. **c,** More than 50% of the events contained pulse-like current modulations: i.e., ~35% of the events presented one modulation, ~14% two modulations, and ~5% three modulations. Nanopore recordings were conducted in 25 mM Tris, pH 7.9, 100 mM NH_4_Cl, and 2 M LiCl at 25 °C. Reprinted with permission from reference [84].

Ideally, the signature for DNA damage detection is that such damage can introduce both event duration and current level changes. By the labelling of aminomethyl-[18-cown-6] (18c6) to the OG sites and optimizing of the electrolyte, detecting and quantification of OG sites in the long a Long hTelo Sequence (120-mer) after exposure to ^1^O_2_ was achieved. The labeled OG yielded a pulse-like signal (signature) in the nanopore current profile (current *vs* time) when the DNA strand was pulled through the α-HL nanopore by the applied voltage (Figure 6). Identification and detection of telomeres for OG utilizing the nanopore is an innovative approach that is envisioned to simultaneously allow quantification of OG and determination of telomere length in one single experiment. The principles described here can be applied to research surrounding the oxidative stress and telomere attrition observed in different diseases including prostate cancer and diabetes [84].

### 4.2. Detection of abasic site in the β-barrel site of the nanopore

DNA abasic (AP) sites are one of the most frequent lesions in the genome derived from either spontaneous hydrolysis or the enzymatic removal of modified bases by glycosylase. AP sites could induce strand breaks and transcriptional mutations resulting in cellular dysfunction [85,86,87,88]. AP site detection has attracted tremendous interest, and many methods have been developed, like fluorescence measurements [89,90], atomic force microscopy (AFM) [91], mass spectrometry [92], ELISA-based assay [93], utilization of aldehyde-reactive probes [94], metalloinsertors [95], and isotope labels [96]. Though, detection methods that can detect multiple sites are still in urgent need [97].

**Figure 7.**
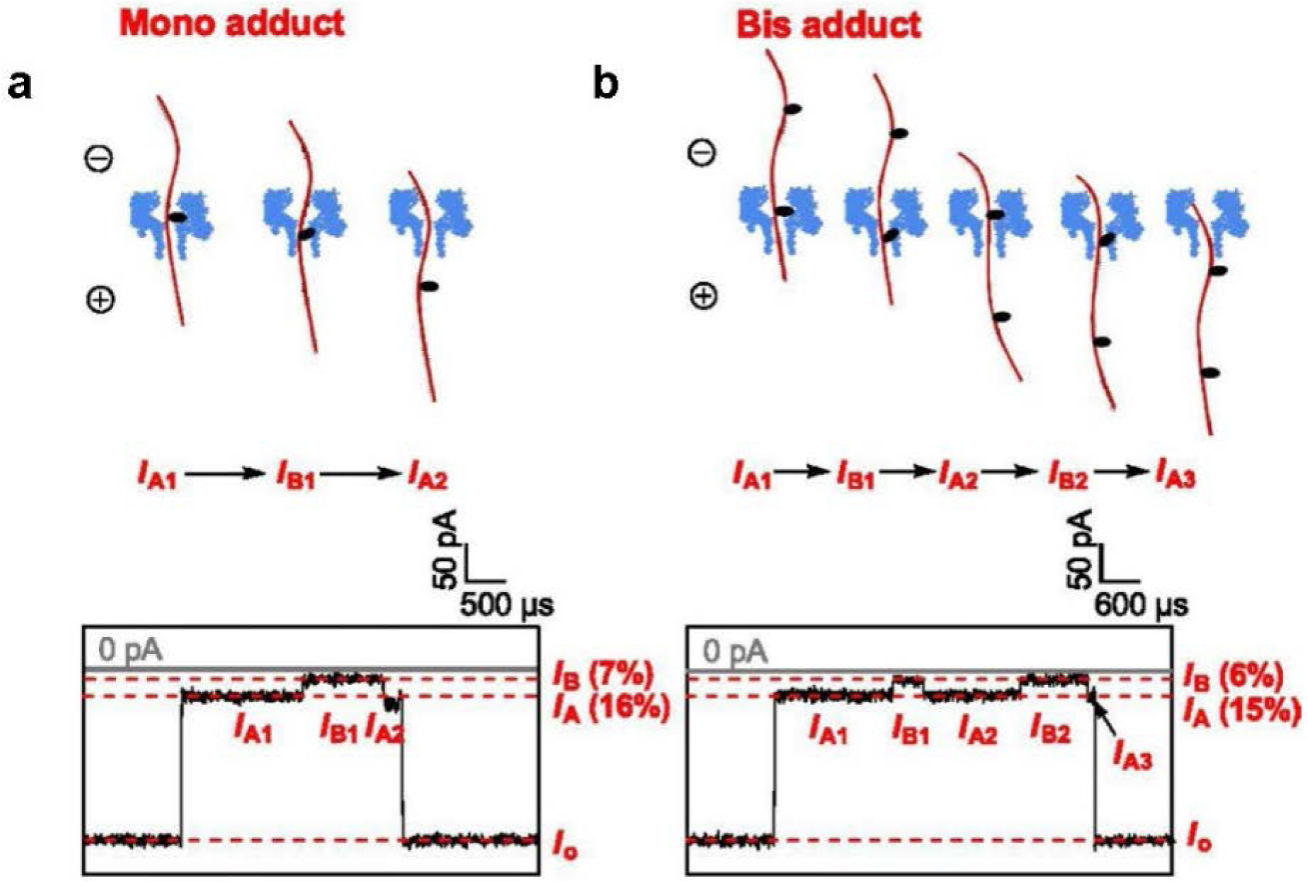
Detection of abasic site in the β-barrel of the nanopore. Individual *i-t* traces of AP-18c6 in homopolymeric strands. **a,** Identification of single AP-18c6 adduct. Sample *i-t* traces for mono adduct (120 mV *trans* vs. *cis*). **b,** Identification of two AP-18c6 adducts. Sample *i-t* traces for bis adducts (120 mV *trans* vs. *cis*). DNA was captured into the nanopore from 5’ entry. The pulse like nanopore signature for AP-18c6 was generated for the AP detection. Reprinted with permission from reference [98].

In the α-HL, studies have demonstrated that the 18c6 labeled abasic site (AP-18c6) can be detected and identified in single-stranded DNA in a NaCl electrolyte solution, which generated a diagnostic pulse-like current signature in the nanopore when the AP-18c6 strand interacted with the β-barrel (Figure 7). It is possible that 18c6-Na^+^ hesitated at the protein constriction and prevented the movement of the DNA molecule. However, after the dissociation of the Na^+^, the 18c6 adduct passed through the constriction site, and a deeper current blockage was generated. Finally, when the DNA had passed through the nanopore, the current returned to its open current level. Most importantly, this method was able to detect multiple AP sites in one DNA strand [98]. Applying the same chemical strategy combined with the help of lesion-specific glycosylases, researches can envision that a variety of DNA base damages and mismatches can be specifically converted to AP sites and then functionalized to the AP-18c6 adduct, allowing their sequence-specific detection in single molecules. This method has the potential to be used for disease/cancer detections caused by an unrepaired AP site.

### 4.3. Abasic site detection in the latch zone of the nanopore

**Figure 8.**
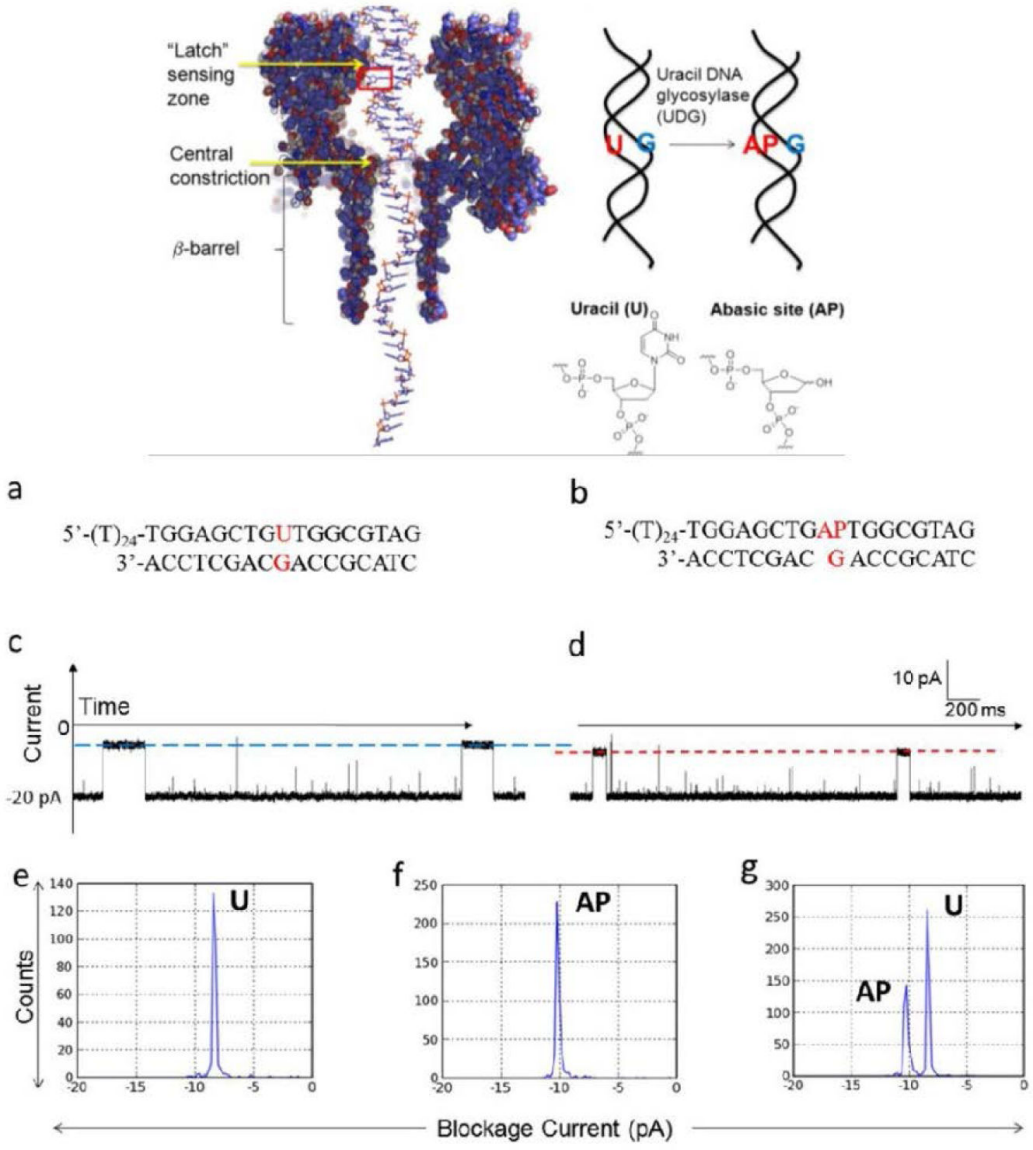
Abasic site detection in the latch zone of the nanopore by monitoring the UDG enzyme activity for dsDNA in the nanopore. Top scheme: Left: The structure of dsDNA with a 5’- poly(T)_24_ tail within WT α-HL. The box indicates the location of the uracil (U) base or the abasic site (AP). Right: Scheme of the UDG hydrolysis reaction. The α-HL structure was taken from pdb 7AHL. DNA structure is shown on a 1:1 scale with α-HL. **a,** Sequence of the starting material formed by a 41-mer U-containing strand hybridized to a 17-mer strand. **b,** Sequence of the product containing AP. **c, d,** Sample current-time (*i-t*) traces for blockages generated by the U duplex (c) or the AP duplex (d) in individual experiments. The blue and red lines indicate the current blockage levels used to determine the duplex identity. **e, f, g,** Histograms of current blockage levels for the U duplex (e), AP duplex (f) and for a mixture of U and AP duplexes (g, mole ratio ~2:1). Single-nucleotide recognition was achieved between the U-containing duplex (a, c, and e) and the AP-containing duplex (b, d, and f) based on a ~2 pA difference in blockage current levels of the unzipping events in a 14 μM DNA, 150 mM buffered KCl solution at −120 mV. Reprinted with permission from reference [99].

The α-HL nanopore has been employed for DNA sequencing, various single-molecule detections, and bimolecular interactions by utilizing its narrowest constriction site, since it has always been considered as the sensing region of the pore. Interestingly, the latch zone, i.e., the vicinity of the vestibule constriction of the pore was recently found to constitute a sensing region as well. This previously unrecognized sensing region was found to be able to detect individual abasic sites in dsDNA in 2013 [99] (Figure 8). Later, further research was carried out which obtained the optimized experimental conditions for identifying the base modification [100].

Studies have successfully demonstrated that U and AP sites can be discriminated in the nanopore by monitoring uracil-DNA glycosylase (UDG) enzyme activity. The UDG hydrolysis reaction converts a uracil (U) base to an AP site (Figure 8). When the dsDNA is trapped at the nanocavity, and the U-G or AP-G pairs are placed at the vicinity of the latch zone of the pore, they can generate distinguishable current levels. During the hydrolysis reaction, the two current levels can be observed at the same time due to partial conversion of U to AP (Figure 8g). This study also provides a guideline for developing new approaches for monitoring enzymatic activity on DNA bases [99]. This newly discovered sensing zone at the latch zone suggests the possible development of new approaches to detect site-specific changes in dsDNA structure relevant to genetic, epigenetic and medical diagnostic applications. Protein mutagenesis that could alter the amino acids at the latch region can possibly change the size and dimension of the channel and further change the chemical and physical interactions between the channel wall and DNA. It is therefore potentially useful in the future to enhance detection specificity and sensitivity.

## 5. Cytosine Modifications Detections

Silver ions specifically interact with C-C mismatches [101–104], while mercury ions specifically interact with T-T mismatches [105–108]. These interactions that strongly stabilize DNA duplexes have been extensively studied recently [109]. Considering that cytosine (C) modifications such as 5-methylcytosine (mC) and 5-hydroxymethylcytosine (hmC) are important epigenetic markers associated with gene expression and tumorigenesis [110–112], we were motivated to explore the interactions of Ag^+^with a DNA duplex containing a single C-C, C-mC, or C-hmC mismatch in the α-HL nanopore. The α-HL has a nanocavity (2.6nm opening with a 1.4nm constriction site) that can capture and hold the DNA duplex, providing an ideal platform for studying both the cations/C-C interaction and how cytosine modifications change the interaction.

### 5.1. Direct and label-free discrimination of cytosine and cytosine modifications utilizing silver ions in the nanopore

In previous studies [113–116], researchers have found that C, mC, or hmC can be recognized by immobilizing the DNA with streptavidin [115] or by chemical modifications [113] in α-HL. When in a solid-state nanopore, it was found that DNA duplexes containing mC and hmC could be discriminated [116], and by using methylated CpG binding proteins [43,114] and chemical modifications via sequencing, the hmC, mC, and C bases could be discriminated as well [117–119].

Recently, studies have shown that cytosine modifications can change the current profiles when the modifications containing dsDNA were trapped in the nanocavity. With the help of silver ions, these DNA modifications could modify DNA stability and lead to changes in event durations. Secondly, these modifications also could alter the DNA space resulting in changes of current levels. This was because mC has only an extra methyl group, while hmC has an extra methyl group and a hydroxy group, which blocked more ion current when these modifications were placed at the latch zone of the nanopore [120].

**Figure 9.**
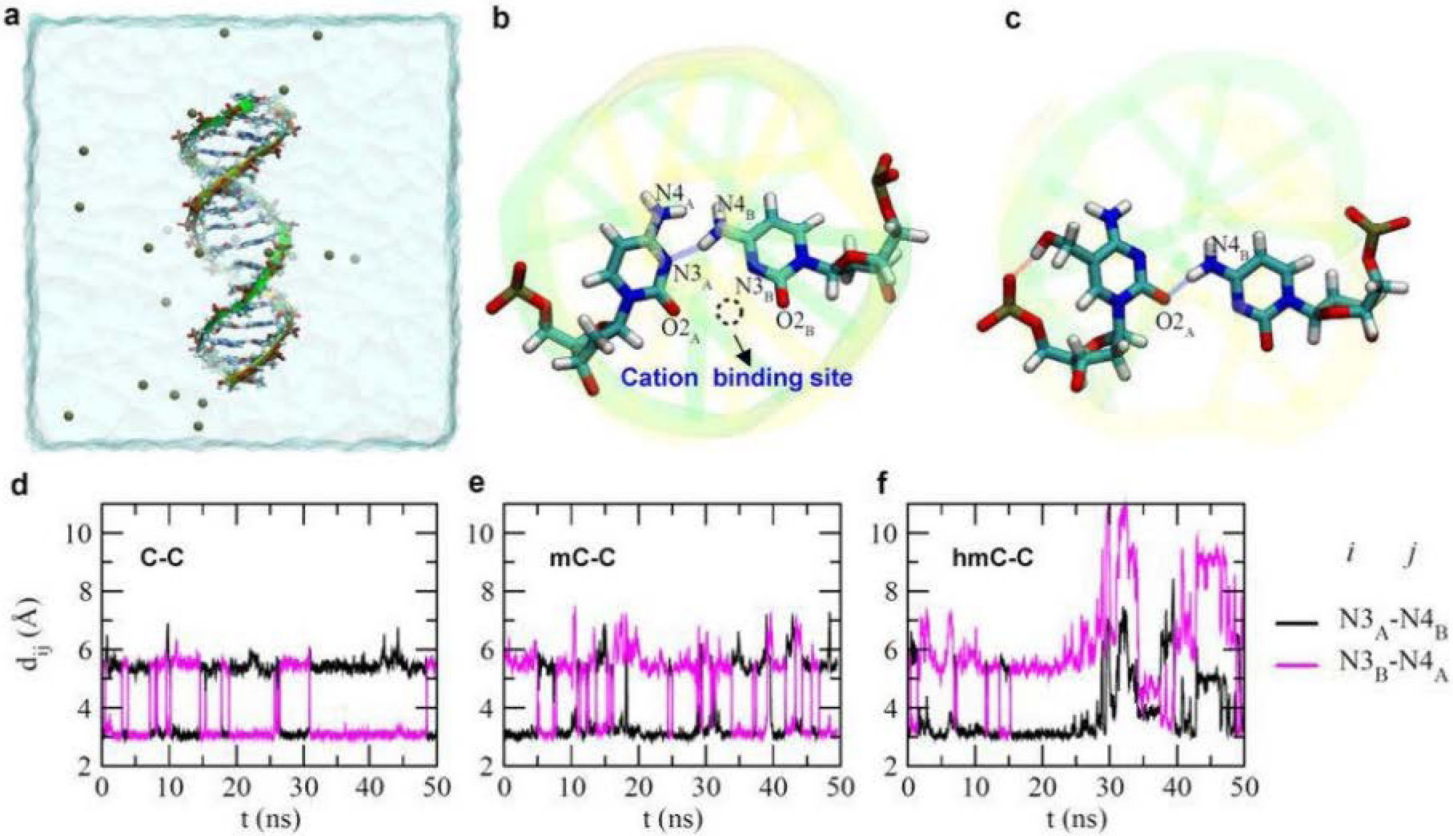
Molecule dynamic simulations revealed a cation binding site in the C-C mismatch and unstable hmc-C pairing. The DNA duplex is in the “stick” presentation, and the two backbones are illustrated as yellow and green belts respectively. Potassium ions that neutralize the entire simulation system are shown as tan balls. Water in a cubic box (78.5 × 78.5 × 78.5 Å3) is shown transparently. (b) A snap-shot of pairing between two cytosine bases. The dashed circle highlights the binding site for a cation. (c) A snap-shot of hmC-C pairing before the pairing was broken. (d-f) Time-dependent distances between the N3 atom of one base and the N4 atom of the other base, in C-C(d), mC-C(e) and hmC-C(f) mismatches. Reprinted with permission from reference [120].

The results were supported by the melting temperature measurement and molecule dynamic simulations. These interactions that strongly stabilize DNA duplexes have been extensively studied recently [109], but the nature of coordination of Ag^+^ with C-C mismatches is still not clearly understood [104,121–123]. The simulations suggest that the paring, via a hydrogen bond, of a C-C mismatch results in a binding site for cations, such as K^+^ and Ag^+^. It is a dynamic coordination between N3_A_ and O2_B_, or N3_B_ and O2_A_ for C-Ag-C interactions [120,124]. The interactions suggest that the coordination of Ag+ in C-Ag-C complexes may have a different mechanism (Figure 9). This approach might be expanded to investigate other cytosine modifications like 5-formylcytosine (5fC) and other metallo-pair interactions.

### 5.2. Methylation detection by designing a mercury interstrand lock

DNA methylation is one of the most commonly occurring epigenetic events in the human genome. It is a covalent addition of a methyl group to the cytosine ring by DNA methyltransferases [125,126]. Before nanopore detection, a uracil is converted from unmethylated cytosine by bisulfite treatment. The α-HL nanopore studies demonstrated that mercury ions can bind both T-T mismatches and U-T mismatches. The Hg^2+^ binding produces a reversible interstrand lock, called MercuLock, which can enhance the hybridization strength by two orders of magnitude (evidenced by the prolonged event durations). Such MercuLock cannot be formed in a 5-methylcytosine-thymine mismatch (mC-T). Therefore the nanopore can be used to distinguish single bases between uracil and 5-methylcytosine in a sequence [127].

**Figure 10.**
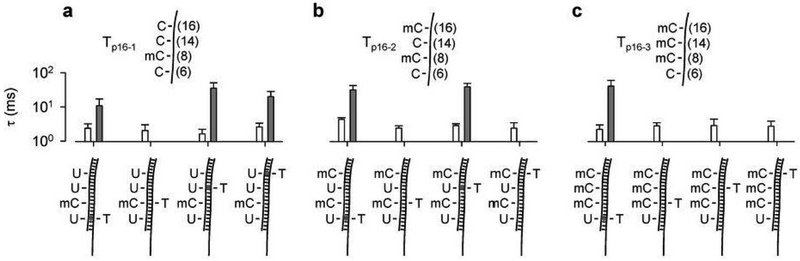
Methylation detection by designing a mercury interstrand lock. (a), (b) and (c) compared the duration of short and long signature blocks for targets Tp16-1 (a), Tpl6-2 (b) and Tpl6-3 (c) detected by four probes PC6, PC8, PC14 and PC16. The duration of signature blocks allowed determining of the methylation status for each of four CpG cytosines. The DNA sequences of the three p16 fragments containined bases 1, 2 and 3 mC in the DNA strand. C can be converted to U by bisulfite treatment and then form a U-T pair which can be stabilized by a mercury ion evidenced by the prolonged duration (gray bar). However, mC can not be converted, so it forms a mC-T pair which can not be stabilized by a mercury ion, therefore only short blocks were observed (white bar). All traces were recorded at +130 mV in 1 M KCl and 10 mM Tris (pH 7.4). Reprinted with permission from reference [127].

More importantly, the study demonstrated the methylation analysis of multiple CpGs in a p16 gene CpG island. Different numbers and distributions of methylated cytosines in the segments of p16 gene can be successfully detected (Figure 10). This work provides a powerful biophysical tool to explore metal ion-nucleic acid interactions in living organisms and in humans. For example, whether Hg^2+^ binds to T-T or a U-T mismatched pair which can compromise the DNA repair process in humans, especially during tumorigenesis, needs further study. This work opens an avenue to the application of metal ion-nucleic acid interactions in rapid detection of single nucleotide alteration in gene sequences, such as pathological point mutations, single nucleotide polymorphism (SNPs), and DNA methylation in variety of disease states including cancer.

## 6. Nanopore Devices

The successful development of a nanopore device platform will rapidly advance both basic and clinical research. With minimum sample preparation, an easy learning and handling curve, and real time testing and analyzation of experimental data, the nanopore device can be used by a doctor for early diagnosis, staging and monitoring of cancer and for observing effectiveness of disease and cancer related drug treatments. This is due to the many disease/cancer biomarkers that exist, like circulating nucleic acids (such as microRNA, DNA) [128–130], peptides [131–133], and proteins [134–137], DNA damages and DNA mutations which can all be detected by the nanopore. Lots of effort is being put in to create a portable and robust nanopore device using solid state nanopores [138–141], protein nanopores [142–144], and α-HL nanopores [145–148] for vast breadth of applications. Through the detection and identification of cancer biomarkers, many academic research and clinical application problems can be solved, which would help to advance modern health care and economy.

The Minion (Oxford Nanopore Technologies) is a commercial portable genome sequencing instrument containing nanopores embedded within a synthetic membrane [149]. It has been used to generate a bacterial genome dataset [150], identify the chromosomal insertion site of a composite antibiotic resistance island [151], and assemble a genome [152,153]. This device is capable of sequencing long DNA fragments (>10 kb) without amplification.

## 7. Conclusion

Biomedical diagnosis for cancer research have attracted great interests for both scientists and physicians, utilizing different kinds of techniques like biophotonics [154], nanoparticles [155], computational intelligence [156], confocal Raman and quantitative phase microscopy [157], nanowire field-effect transistor [158], optical imaging [159], metabolic fingerprinting [160], fluorescence spectroscopy [161] and so on. A steady stream of money flows into endeavors to apply whole genome and sequencing to the development of patient-specific treatment strategies and personalized medicine.

With newly emerged nanopore sequencing technologies, devices like Ion Proton (California-based Life Technologies), HiSeq (San Diego-based Illumina), and Swiss giant Roche, a frontrunner in personalized healthcare, have made huge contributions to the sequencing technology field. Competition between companies has been able to push fast, accurate, and low-cost sequencing into the clinical space and market. Another growing trend in the field of nanopore research is the development of sensors for biodetection of intramolecular and intermolecular interactions as we discussed above. The detection of these targets using the nanopore has advanced considerably over the past ten years [46], and this has led to promising developments of new bioanalytic and diagnostic tools [58]. Unfortunately, the low physiological concentrations of many of these biomarkers [162–165] can dramatically lower the rate at which a target molecule enters the nanopore. This requires the enrichment of the target and limits the practical applications of nanopore-based sensors [165]. All nanopore sensors are facing two common challenges which are low detection efficiency and fast translocation speeds [166].

Enhancing the capture rate and slowing down the translocation speed at the same time are seemingly contradictory actions, but promisingly, the transmembrane salt gradient has proven to be an effective approach for enhancing the capture of double-stranded DNA (dsDNA) and reducing their translocation speed in a synthetic nanopore [167]. Several theoretical studies [168–170] were performed to explore the principles behind this phenomenon, and they suggest that osmotic water flow, electro-osmotic flow (EOF), and electrophoresis may each play their own role. However, these calculations without experimental data were focused on a synthetic pore system, and their results were still being debated. More efforts are urgently needed in this direction of study for developing versatile and accurate nanopore sensors. Another challenge for the application of a nanopore in clinical detections is identifying the target in a mixture environment and discriminating the target from its background molecules. Reliable nanopore signatures are required for this accurate detection. These nanopore signatures have both a specific event duration and a current level change compared to the background signals.
